# Prevalence of Extrapyramidal Symptoms in In-Patients With Severe Mental Illnesses: Focus on Parkinsonism

**DOI:** 10.3389/fneur.2020.593143

**Published:** 2020-11-10

**Authors:** Beatrice Roiter, Giorgio Pigato, Angelo Antonini

**Affiliations:** Department of Neuroscience, University of Padova, Padova, Italy

**Keywords:** psychiatric inpatient, Parkinson's disease, dopamine replacement therapy, extrapyramidal symptoms, DaTscan SPECT

## Abstract

Patients with severe mental illnesses may present extrapyramidal symptoms as part of a concomitant neurological disorder or secondary to medications. Extrapyramidal symptoms are frequently unrecognized, have negative consequences for adherence to treatment, negatively affect quality of life and can induce stigma. We estimated and correlated with demographic and clinical variables prevalence of extrapyramidal symptoms in in-patients with severe mental illnesses. Additionally we evaluated ^123^I-FP-CIT SPECT binding to striatal dopamine transporter in subjects with clinical manifestations suggestive of Parkinson's Disease and recorded therapeutic management and clinical evolution for 6-months. Extrapyramidal symptoms were present in 144 out of 285 patients (50.5%), mainly tremor (94 patients, 33%). There were 38 patients (13.3%) with parkinsonism and they had older age, more medical comorbidities and medical treatments. In 15/38 patients striatal dopamine transporter binding was abnormal resulting in dose reduction or change of psychotropic drugs as well as combination with antiparkinson therapy. Our study confirmed the clinical and epidemiological relevance of extrapyramidal symptoms among inpatients with severe mental illnesses. A small percentage of patients with extrapyramidal symptoms had features compatible with possible diagnosis of Parkinson's Disease. ^123^I-FP-CIT SPECT was useful to identify dopaminergic dysfunction and initiate dopamine replacement therapy.

## Introduction

Patients with Severe Mental Illnesses (SMI) can present Extrapyramidal Symptoms (EPS) as part of primary neurological disorders or following psychotropic treatment. EPS are linked to lower treatment adherence, poorer psychiatric prognosis and increased mortality (1, 2) and are more severe and frequent in patients with SMI than in other psychiatric disorders, with higher prevalence (68–74%) among hospitalized patients (3, 4). Patients with bipolar disorder are particularly likely to develop Parkinson's Disease (PD) (5).

First Generation (FGAs) more than Second Generation Antipsychotics (SGAs) are drugs with the highest risk to trigger drug-induced parkinsonism (DIP) (6–8), but also Selective Serotonin Reuptake Inhibitors (SSRI), lithium salts and valproate can occasionally induce EPS (9–11).

Among EPS, parkinsonism is particularly challenging because it may unmask a neurodegenerative disease. Recent studies in patients chronically exposed to antipsychotics (APS) have shown, in some individuals, loss of dopamine nerve terminals with ^123^I-FP-CIT SPECT and benefit from levodopa replacement therapy (12–14). However, these studies regarded almost exclusively patients with schizophrenia.

We aimed at assessing prevalence of EPS in psychiatric inpatients and at evaluating prospectively clinical outcome of patients with parkinsonism, particularly in relation to possible PD.

## Materials and Methods

We performed two monocentric observational studies: the first was cross-sectional and regarded all psychiatric inpatients compared for presence/absence of EPS; the second was prospective and focused on patients with Parkinsonism. Both studies were approved by our Local Ethics Committee (CESC Padova, AOP0300-DDMEDP-3323/AO/14), informed consent was obtained after all procedures had been fully explained and all data have been stored in our Department.

Our objectives were: to evaluate prevalence rates of whole as well as of single EPS in subjects consecutively admitted to our Inpatient Psychiatric Unit during a period of 18 months; to test for significant association between EPS and demographic (age, gender) and clinical variables (psychiatric and neurological family history, medical comorbidities, psychiatric diagnosis and duration of psychiatric disorder); to evaluate clinical outcome of patients with SMI and features suggestive of PD during a 6-month follow up. Patients discharged after short hospital stay (≤4 days) were excluded from the study.

Psychiatric diagnosis was made according to Diagnostic and Statistical Manual of Mental Disorders (DSM 5) criteria through a semi-structured clinical interview (MINI International Neuropsychiatric Interview DSM 5). Current medications were recorded.

A complete neurological examination was carried out and standardized through a predefined form enriched by items of the Unified Parkinson Disease Rating Scale part III and of DSM 5 to establish the presence of EPS including dystonia, akathisia, dyskinesia, tremor and parkinsonism. Particularly we included items of UPDRS about tremor, rigidity, finger tapping, leg agility, gait and posture. Diagnosis of dystonia, akathisia, dyskinesia and tremor were based on DSM 5 definitions and in line with the indications from the Movement Disorders Society. We listed separately the presence of motor abnormalities, such as postural instability, mild alterations of gait, isolated bradykinesia and isolated extrapyramidal rigidity which we named “non-specific extrapyramidal alterations.”

Patients with parkinsonism and clinical features which may have suggested PD (asymmetry, predominant tremor over rigidity and bradykinesia, no other drug-induced movement disorders) (15, 16) underwent ^123^I-FP-CIT SPECT. ^123^I-FP-CIT SPECT was evaluated by a specialist in nuclear medicine blind to the clinical condition of the patient and defined as abnormal or normal.

These patients were first managed with targeted interventions based on clinical judgement: watchful monitoring, reduction of APS or switching to a different APS with less propensity to induce EPS. Patients with scan abnormalities in case of low or absent reduction of parkinsonian symptoms at 1st month were treated with levodopa.

A clinical evaluation at baseline and at 1, 3, and 6 months follow up was performed for all patients by Clinical Global Impression Severity/Improvement Scale for parkinsonism (CGI-S parkinsonism and CGI-I parkinsonism) and for psychiatric symptoms (CGI-S psychiatric disease and CGI-I psychiatric disease).

Cases with uncertain EPS were video-recorded and discussed with a neurologist with expertise in movement disorders (AA) who also screened all cases with parkinsonism, reviewed all ^123^I-FP-CIT SPECT by visual-qualitative-method and managed patients during the follow up.

## Statistical Analysis

We estimated prevalence rates of EPS overall and of every EPS.

A univariate statistical analysis was performed to find association between EPS and all above-mentioned demographic and clinical variables by chi-square test or Fisher exact test (for qualitative variables) and by Wilcoxon rank sum test (for quantitative variables).

Statistical significance was assumed if *p* ≤ 0.05.

CGI scores were described by median scores. We did not perform statistical analysis because of the small size of the subsample.

## Results

The sample consisted of 285 patients out of 325 admitted in the 18 months of the study. Data were missing in 40 patients because of short hospital stay or lack of consent.

Mean age was 46 ± 17 years, with normal distribution and range between 14 and 92 years. Gender was equally distributed (50% male). About half of patients (51.6%) had familiarity for psychiatric disorder, 16.5% had familiarity for EPS, 60% had medical comorbidities (14% neurological, 20% endocrine-metabolic, 26% cardiovascular, and 37% others). Regarding psychiatric diagnoses, 26.7% had psychosis, 27.7% bipolar disorders, and 26% major depression. Other diagnoses (isolated or as psychiatric comorbidity) were personality disorders (17.8%), substance use disorders (14.4%), and intellectual disabilities (3.2%). Mean duration of psychiatric disease was 33 ± 18 years. The majority of patients (75.8%) were treated with APS (5.6% FGAs, 71% SGAs), 41.8% with mood stabilizers (22.5% valproic acid, 9.1% lithium salts, and 10.2% other anticonvulsants), 46% with antidepressants (24.2% SSRI, 1.4% tricyclic, and 20% others), and 66.3% with benzodiazepines. A very small portion of patients (3.9%) were on anticholinergic or dopaminergic drugs. Polypharmacy was given in most cases. About half of the sample was given additional non-psychotropic drugs, but none of them were given medications at risk for inducing EPS (antiemetics, calcium-channels inhibitors, chemotherapics, immunosuppressants, and antimalarials) (9, 17–19). Characteristics of the sample are summarized in Table 1.

**Table 1:** Demographic and clinical variables.

Total sample (N)	325	
Final sample	285	
	Mean (SD)	Median [25^°^,75^°^ percentile] min:max
Age (years)	46 (17)	45 [33, 56] 14:92
	N	%
Gender (male)	143	50
Familiarity for psychiatric disorder	147	51.6
Familiarity for EPS	47	16.5
Medical comorbidity (any)	171	60
Neurologic	40	14
Endocrine	57	20
Cardiovascular	74	26
Other	105	37
Medical treatment (any)	143	50.2
Dopaminergic/anticolinergic	11	3.9
Drugs inducing EPS	0	0
Psychiatric disorder		
Schizophrenia and other non-affective disorders	76	26.7
Bipolar disorder	79	27.7
Depressive disorder	74	26
Personality disorder	51	17.8
Substance abuse	41	14.4
Intellectual disability	9	3.2
Others	35	12.3
	Mean (SD)	
Duration of psychiatric disorder (years)	33 (18)	
Current psychiatric treatment	N	%
APS	216	75.8
FGAs	16	5.6
SGAs	202	71
Mood stabilizers	119	41.8
Lithium salts	26	9.1
Valproate	64	22.5
Others	29	10.2
Antidepressants	131	46
Tricyclics	4	1.4
Selective Serotonine Reuptake Inibitors	69	24.2
Others	57	20
Benzodiazepines	189	66.3


**Figure 1:**
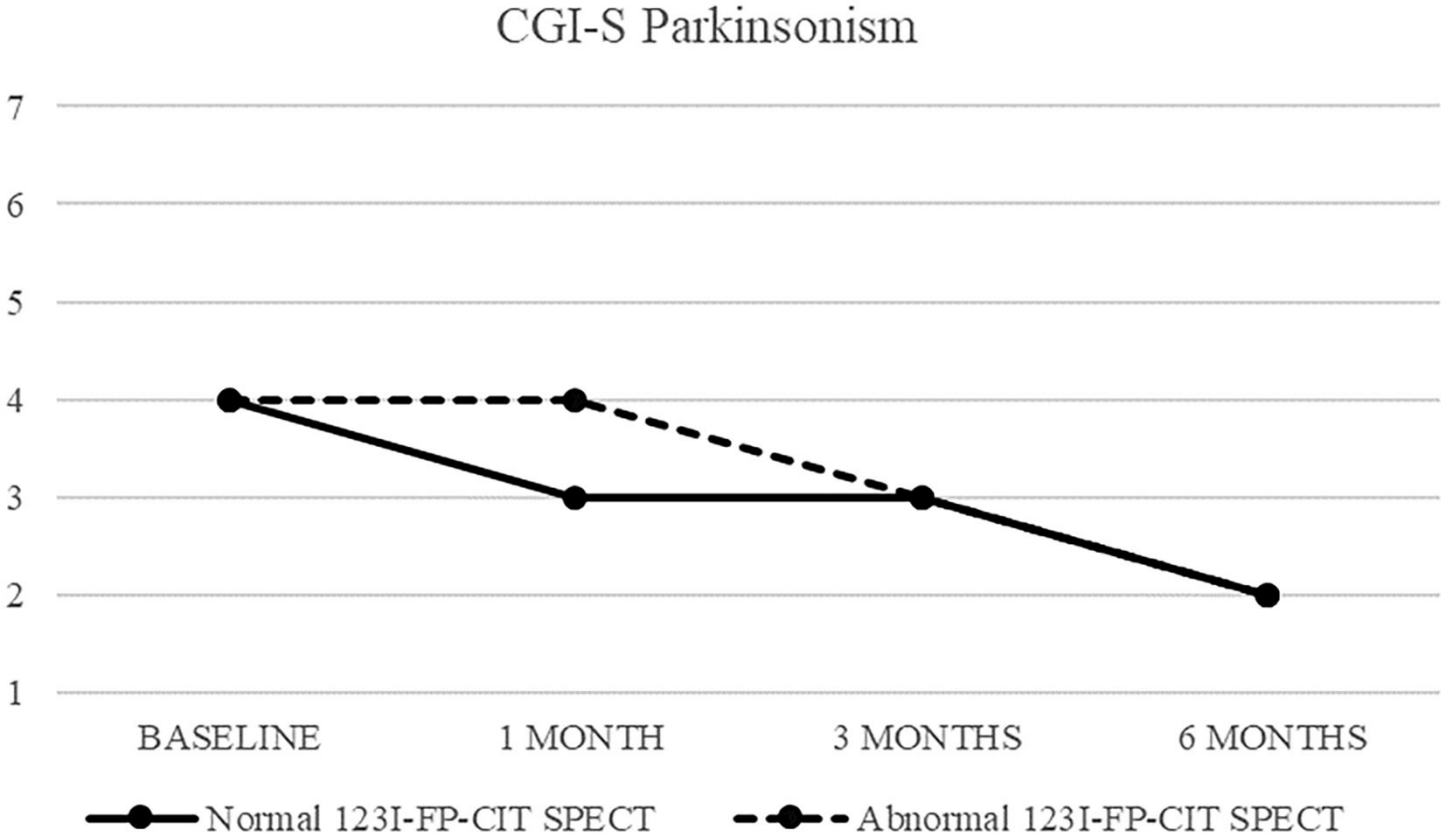
CGI-S of parkinsonism in patients with normal (continuous line) and abnormal (dashed line) ^123^I-FP-CIT SPECT.

Prevalence of EPS overall was 50.5% (144 cases). The most frequent EPS was tremor (94 cases, 33% of the total sample, and combined form in most cases). The second EPS was parkinsonism (38 cases, 13.3%). Other EPS showed the following prevalence rates: akathisia (2.1%), tardive dyskinesia (1.4%), and acute dystonia (0.7%) (Figure 1). Patients with uncertain motor alterations (20.2%) were not considered in the overall prevalence. The two most frequent motor symptoms were isolated bradykinesia (18%) and isolated extrapyramidal rigidity (10%).

We compared patients with and without EPS for groups with sufficient sample size: “EPS overall,” “tremor” and “parkinsonism.” We found no differences between patients with and without “EPS overall.” Patients with and without parkinsonism showed significant differences in age (respectively, median age 60 [48, 69] vs. 44 [31, 54], *p* = 0.001), medical comorbidity (respectively, 82% vs. 58%) (*p* = 0.01) and medical therapy (respectively, 86% vs. 46%) (*p* = 0.0001). Ongoing treatment with FGAs was significantly different in patients with and without EPS overall (respectively, 72.7% vs. 27.3%) (*p* = 0.04) and with and without parkinsonism (respectively, 77.3% vs. 40.2%) (*p* = 0.03). Patients with and without tremor showed significant differences in ongoing treatment with mood stabilizers (respectively, 59% vs. 40%) (*p* = 0.04). These results are summarized in Table 2.

**Table 2:** Comparisons between patients with and without EPS, with and without tremor and with and without parkinsonism.

			**EPS overall**			**Tremor**			**Parkinsonism**		
			**yes**	**no**	***p***	**yes**	**no**	***p***	**yes**	**no**	***p***
Age (year)		Median	47	44	ns	46	45	ns	60	44	0.001
		25°, 75° perc min-max	36, 57 14-92	31, 55 15-83		37, 56 15-92	31-57 14-84		48, 69 25-82	31, 5414-92	
Gender		% male	50.3	50	ns	50	50.3	ns	42.8	51	ns
Familiarity for psychiatric disorder		%	48.2	55.5	ns	47.7	53.6	ns	44	52.4	ns
Familiarity for EPS		%	18.4	14.2	ns	20.7	14.4	ns	17.4	16.4	ns
Medical comorbidity	Any	%	65.7	55.2	ns	64.1	59.1	ns	82.1	58.3	0.014
	Neurological	%	17.9	10.4	ns	16.3	13.5	ns	28.6	13	ns
	Endocrine	%	22.1	19.1	ns	23.9	18.9	ns	32.1	19.4	ns
	Cardiovascular	%	26.2	26.9	ns	27.2	26.2	ns	39.3	25.1	ns
Medical therapy		%	55.2	44.4	ns	57.6	46.3	ns	85.7	46.1	0.0001
Psychiatric disorder	Schizophrenia and other psychosis	%	27.2	25.8	ns	27.7	25.9	ns	35.7	25.5	ns
	Depressive Disorders	%	25.8	25.8	ns	21.3	28.2	ns	39.3	24.3	ns
	Bipolar Disorders	%	27.2	28.1	ns	25.5	28.3	ns	14.3	29.2	ns
	Personality Disorders	%	18.4	17.2	ns	21.3	16.0	ns	3.5	19.4	ns
	Substance abuse	%	12.9	16.4	ns	12.8	15.5	ns	10.7	14.9	ns
	Others	%	12.9	11.7	ns	17	9.9	ns	14.3	12.2	ns
APS (any)		%	54.1	46	ns	64	55.1	ns	89	60	ns
FGA		%	72.7	27.3	0,04	59.1	41	ns	77.3	40.2	0,03
SGA		%	52.8	47.2	ns	64.6	39.5	ns	90.8	85.3	ns
SSRI		%	58.2	41.8	ns	62.7	50.2	ns	87.3	75	ns
MOOD STABILIZERS		%	59.1	40.8	ns	59.1	40.9	0.04*	70	65.1	ns
Duration of psychiatric disorder (years)		Median	28	27	ns	23	28	ns	33	27	ns
		25°, 75°	19, 40	20, 42		18, 40	20, 40		20, 60	19, 40	

Twenty-six patients (68.4% of patients with parkinsonism) showed clinical features suggestive of PD and underwent ^123^I-FP-CIT SPECT to confirm presence of striatal dopaminergic deficits. This group was characterized by almost equally distributed sex (52% males), age over 50 years (range 51–81), mean age 65 (±9) years, prevalence of affective (65.4%) over psychotic disorder, and long duration of psychiatric illness (mean duration 23.2 ± 16.8 years). At baseline, all 26 patients were taking APS (SGAs: 73%, FGAs: 27%), 9/26 cases in combination with mood stabilizers (lithium salts or valproic acid). Eleven out of 26 patients (42.3%) had normal ^123^I-FP-CIT SPECT striatal binding. Nonetheless in four cases it was decided to gradually reduce dose or switch to another APS (clozapine).

Fifteen patients (57.7%) showed abnormal ^123^I-FP-CIT SPECT striatal binding (mostly limited to the posterior putamen) and 12 of them were prescribed either levodopa (11 cases) or pramipexole (one case) 1 month after APS reduction.

**Figure 2:**
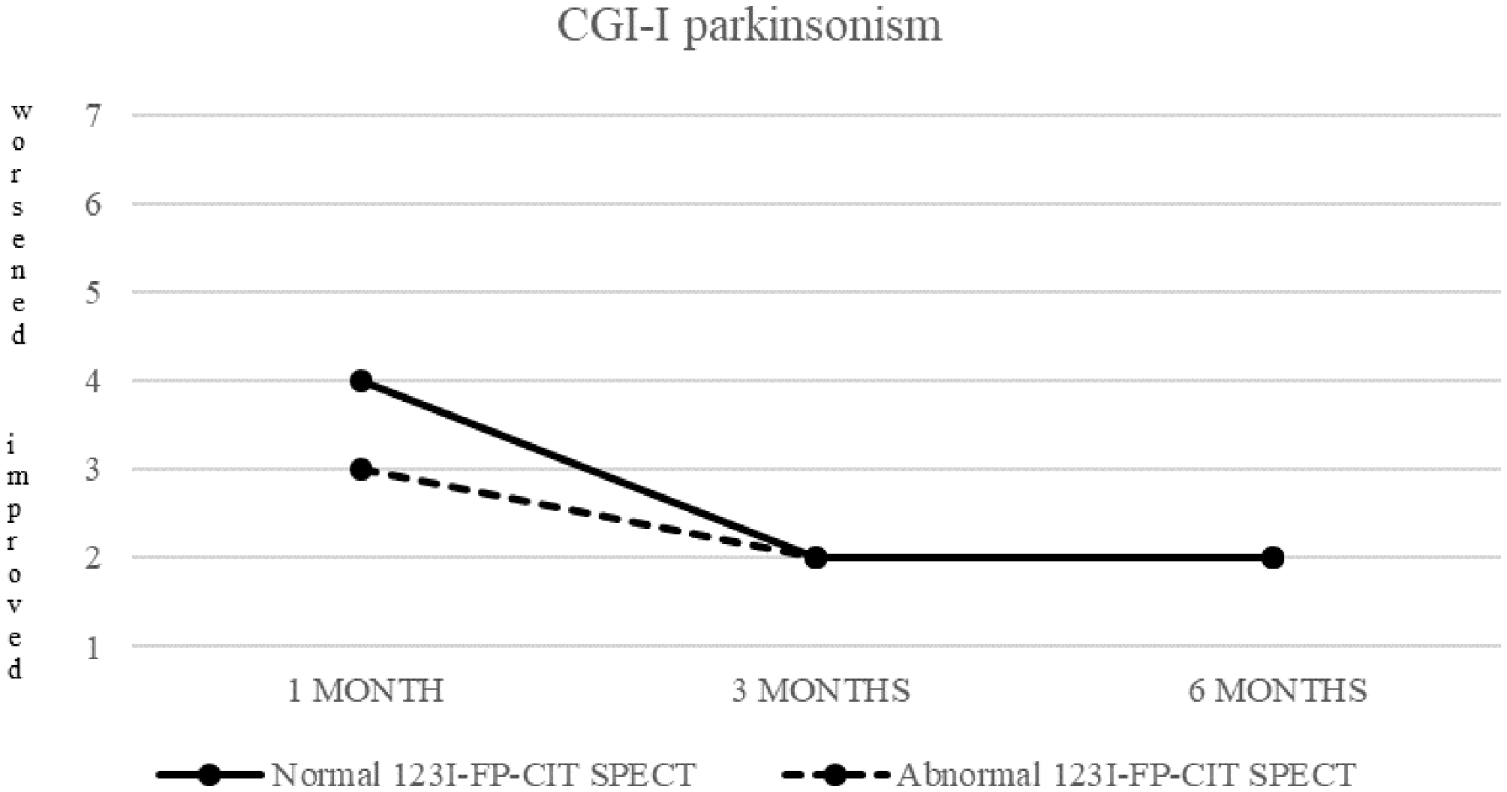
CGI-I of parkinsonism in patients with normal (continuous line) and abnormal (dashed line) ^123^I-FP-CIT SPECT.

Clinical response to treatment of these 26 patients is showed in Figure 2.

Both patients with normal and abnormal SPECT showed a progressive decrease in median CGI-S-parkinsonism scoring, from four (moderately ill) at baseline to two (borderline ill) at 6 months. Patients with normal SPECT showed no change (median CGI-I = 4) after 1 month, but a significant improvement (CGI-I = 2) after 3 and 6 months, while patients with abnormal SPECT showed improvement already at 1 month follow up.

**Figure 3:**
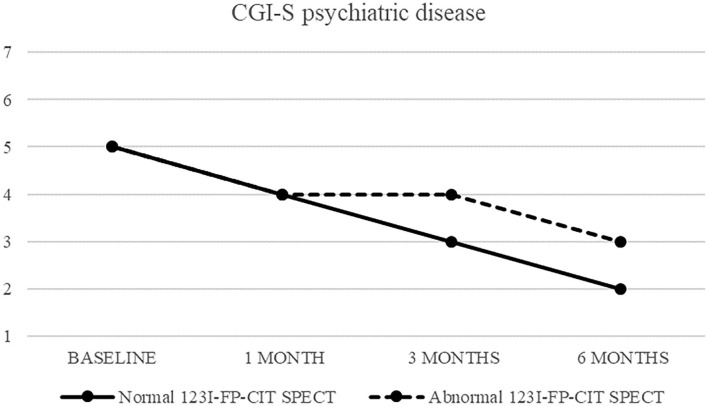
CGI-S of psychiatric disease in patients with normal (continuous line) and abnormal (dashed line) ^123^I-FP-CIT SPECT.

**Figure 4:**
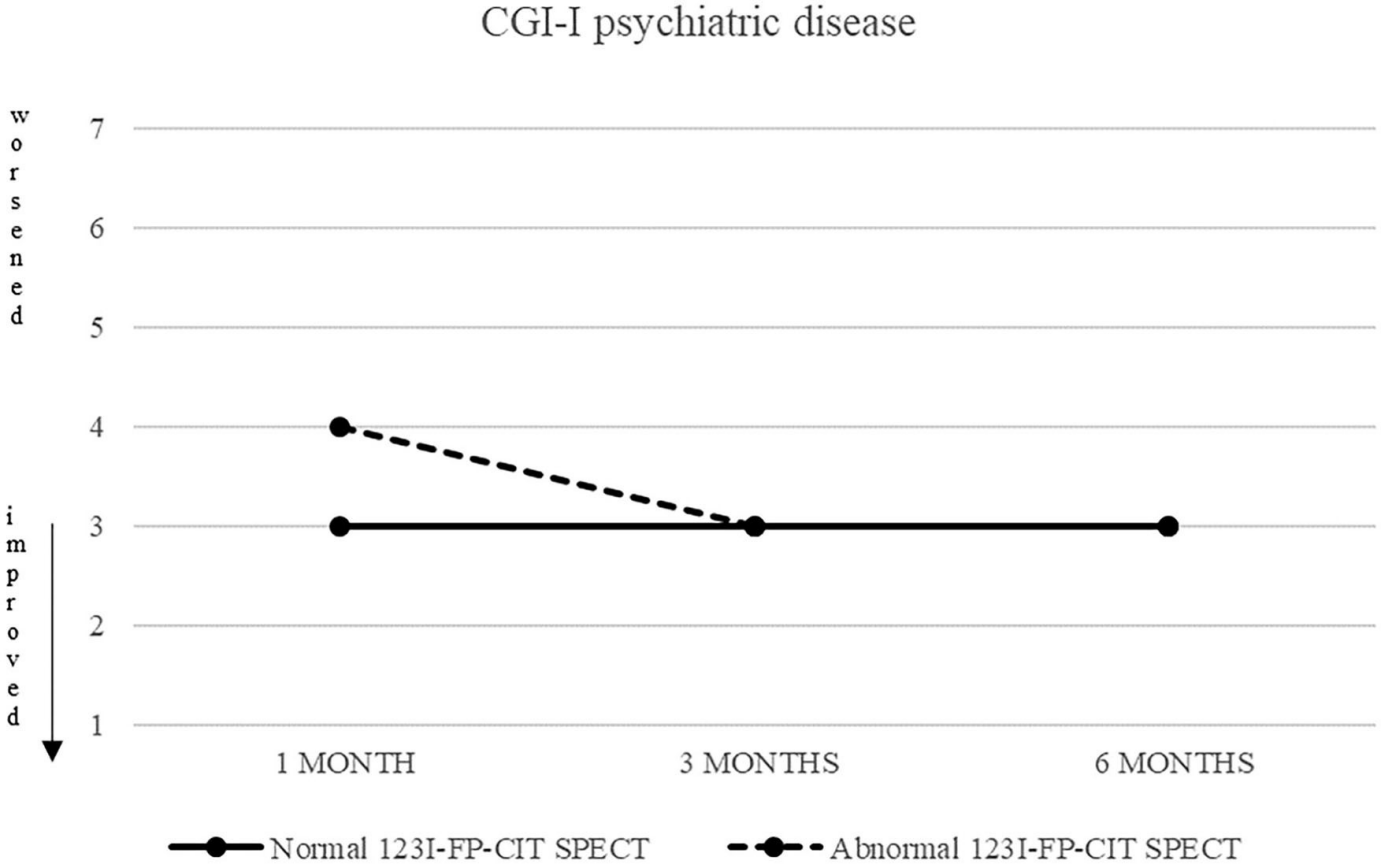
CGI-S of psychiatric disease in patients with normal (continuous line) and abnormal (dashed line) ^123^I-FP-CIT SPECT.

Also CGI-S scoring about psychiatric disease progressively decreased, from five (markedly ill) to two after 6 months. CGI-I confirmed an improvement, although less pronounced (minimally improved after 3 and 6 months in both groups) (Figures 3, 4).

## Discussion

We reported high prevalence of EPS (50.5%), particularly tremor and parkinsonism, in psychiatric patients routinely managed in our Psychiatric Hospital Unit, which is almost double of that reported in other series (28%) (20).

To our knowledge, there are no other studies on overall prevalence of EPS among psychiatric inpatients. Available studies regarded mainly patients with schizophrenia treated with APS, selected for type and time of exposure, or focused on one specific EPS. Other studies found that parkinsonism is often under-recognized and that, in patients treated with APS, its prevalence varies between 20 and 35%, which is similar to that of akathisia and tardive dyskinesia (10–30% and 20–40%, respectively) (15, 21, 22). Prevalence rates of parkinsonism (13.3%), akathisia (2.1%), and tardive dyskinesia (1.4%) was lower in our study than literature.

Discrepancies may be attributed to various reasons. First, we used a structured assessment for detection of EPS in a hospital setting. Second, our sample consisted of a wide range of psychiatric disorders with different propensity to be associated to EPS. Third, most patients were prescribed new generation APS (greater use of SGAs) which have lower risk to induce EPS. Lastly, isolated bradykinesia (17.8% of total sample) or extrapyramidal rigidity (9.8%) were not considered as full-blown EPS according to DSM 5, which may have resulted in slight underestimation of EPS prevalence.

It should be stressed that our cross-sectional design did not allow to identify any causal relationship between all variables considered: evaluation of prevalent cases was a limit of the study because we could not assess past pharmacological treatment. We only reported psychopharmacotherapy in general terms because most patients were taking a long-term polypharmacy, so we were not able to know cumulative doses and times of exposure, both involved in the genesis of EPS. However, significant differences were found in ongoing treatment, particularly higher prevalence of FGAs in patients with EPS overall and parkinsonism compared to patients without, and higher prevalence of mood stabilizers in patients with isolated tremor compared to patients without.

We found that only a minority of patients fulfilled criteria for possible PD and an even smaller proportion presented reduced striatal DAT binding. Patients with parkinsonism were on average 20 years older and had more comorbidities, which is in line with previous studies (23, 24). This might suggest greater vulnerability in elderly subjects but also greater likelihood to develop dopaminergic dysfunction in that age range. It should also be underscored that patients with parkinsonism had a very long duration of psychiatric illness, predominant bipolar disorder as well as exposure to various APS and mood stabilizers over the years. Nonetheless the severity of parkinsonism was moderate at first visit, with some improvement during follow up possibly due to APS dose adjustment or change. In those subjects we introduced primarily levodopa with one exception where we opted for pramipexole since patient presented tremor and apathy. The positive outcome of these patients is in line with previous studies (14).

This study confirmed the clinical and epidemiological relevance of EPS among inpatients with SMI, mainly tremor and parkinsonism. A small percentage of patients with EPS have features compatible with possible PD and presence of reduced striatal DAT binding, resulting in treatment modification.

## Data Availability Statement

The raw data supporting the conclusions of this article will be made available by the authors, without undue reservation.

## Ethics Statement

The studies involving human participants were reviewed and approved by Ethics Committee for Clinical Trial (CESC) - Padova - Prot. AOP0300-DDMEDP-3323/AO/14. The patients/participants provided their written informed consent to participate in this study.

## Author Contributions

BR and GP drafted the work. AA revisited it critically. All authors gave substantial contributions to the conception and design of the work and to the acquisition, analysis or interpretation of data, and provided approval for publication of the content and agreed to be accountable for all aspects of the work in ensuring that questions related to the accuracy or integrity of any part of the work are appropriately investigated and resolved.

## Conflict of Interest

The authors declare that the research was conducted in the absence of any commercial or financial relationships that could be construed as a potential conflict of interest.
